# Corticosteroid-induced spinal epidural lipomatosis in the pediatric age group: report of a new case and updated analysis of the literature

**DOI:** 10.1186/1546-0096-9-5

**Published:** 2011-02-01

**Authors:** Jana C Möller, Randy Q Cron, Daniel W Young, Hermann J Girschick, Deborah M Levy, David D Sherry, Akiko Kukita, Kaoru Saijo, Frank Pessler

**Affiliations:** 1 Division of Rheumatology and Immunology, Children's Hospital, Medical Faculty "Carl Gustav Carus", Technical University Dresden, Fetscherstr. 74, 01307 Dresden, Germany; 2 Department of Pediatrics, Division of Rheumatology, Children's Hospital of Alabama, University of Alabama at Birmingham, Children's Park Place, Ste. 210, 1601 4th Avenue South, Birmingham, AL 35233, USA; 3 Department of Radiology, Children's Hospital of Alabama, 1600 Seventh Avenue, South ACC Building Suite #306, Birmingham, AL 35233, USA; 4 Clinic for Paediatric and Adolescent Medicine, Perinatal Centre of the Vivantes Klinikum im Friedrichshain, Landsberger Allee 49, 10249 Berlin, Germany; 5 Pediatric Rheumatology, Hospital for Sick Children, 555 University Avenue, Toronto, ON M5G 1X8, Canada; 6 Division of Rheumatology, The Children's Hospital of Philadelphia, 3405 Civic Center Boulevard, Philadelphia, PA 19104, USA; 7 Department of Microbiology, Faculty of Medicine, Saga University, 5-1-1, Nabeshima, Saga 849-8501, Japan; 8 Department of Cellular and Molecular Medicine, School of Medicine, University of California San Diego, 9500 Gilman Dr., CMMW (GPL) Rm. 219, La Jolla CA 92093-0651, USA; 9 Department of Infection Genetics, Helmholtz Centre for Infection Research, Inhoffenstr. 7, 38124 Braunschweig, Germany

## Abstract

Spinal epidural lipomatosis is a rare complication of chronic corticosteroid treatment. We report a new pediatric case and an analysis of this and 19 pediatric cases identified in the international literature. The youngest of these combined 20 patients was 5 years old when lipomatosis was diagnosed. Lipomatosis manifested after a mean of 1.3 (+/- 1.5) years (SD) (median, 0.8 years; range, 3 weeks - 6.5 years) of corticosteroid treatment. The corticosteroid dose at the time of presentation of the lipomatosis ranged widely, between 5 and 80 mg of prednisone/day. Back pain was the most common presenting symptom. Imaging revealed that lipomatosis almost always involved the thoracic spine, extending into the lumbosacral region in a subset of patients. Predominantly lumbosacral involvement was documented in only two cases. Although a neurological deficit at presentation was documented in about half of the cases, surgical decompression was not performed in the cases reported after 1996. Instead, reducing the corticosteroid dose (sometimes combined with dietary restriction to mobilize fat) sufficed to induce remission. In summary, pediatric spinal epidural lipomatosis remains a potentially serious untoward effect of corticosteroid treatment, which, if recognized in a timely manner, can have a good outcome with conservative treatment.

## Introduction

Spinal epidural lipomatosis is a rare but well documented untoward effect of chronic corticosteroid treatment that was first described 1975 in an adolescent treated with corticosteroids after a kidney transplant [1]. Besides this iatrogenic etiology, there are idiopathic cases that share adiposity as a risk factor [2,3] and may manifest even in the pediatric age group [4]. Independent of the cause, an overgrowth of fatty tissue in the epidural sac leads to back pain and symptoms of spinal nerve or cord compression, depending on the location and extent of the lesion. Diagnosis is best made by spinal magnetic resonance imaging (MRI), and treatment consists of steroid reduction, which is sometimes combined with dietary restriction of carbohydrate or fat intake to help metabolize the fat. Surgical decompression by laminectomy is reserved for severe cases [2]. The exact pathogenic mechanism of corticosteroid-associated epidural lipomatosis is unclear. Although it likely represents a subtype of iatrogenic Cushing syndrome, it remains to be explained why only a small percentage of individuals on chronic corticosteroid treatment develop an accumulation of fatty tissue at this particular anatomic site. Whereas epidural lipomatosis in adults has been subject to relatively large studies [2], only case reports [1,5-14] and three small series [15-17] have dealt with this entity in the pediatric age group. We now present a new pediatric case of corticosteroid-associated spinal epidural lipomatosis and an updated analysis of the pediatric cases published in the international literature.

## Patients and Methods

### Case report

**Figure 1:**
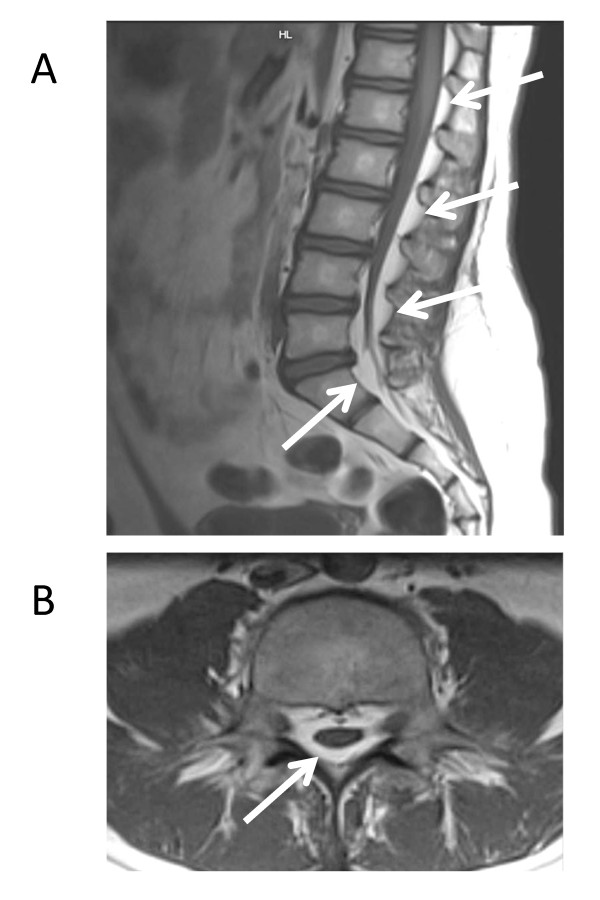
**MRI including the lumbar spine, demonstrating spinal epidural lipomatosis in the presented patient after 4.5 months of treatment with prednisone and methylprednisolone**. The arrows point to selected areas of the pathological accumulation of fatty tissue in the epidural space. This was not present in a baseline MRI performed before initiation of corticosteroid therapy (image not shown). T1 weighted images; A, sagittal plane; B, axial plane.

A 10-year-old Caucasian girl (weight, 36 kg) presented with pain of the ear helices and pain and swelling of several ribs bilaterally. There was a history of chronic bilateral Achilles tendonitis and right knee arthritis. One week later, the patient developed laryngeal pain, and otolaryngologic evaluation revealed a partially paralyzed vocal cord. Taken together, these findings established a clinical diagnosis of relapsing polychondritis. The patient was also noted to have bilateral sacroiliac (SI) joint tenderness, and MRI revealed SI joint synovitis. Treatment with methylprednisolone (1 g/d i.v. × 3 d), prednisone (1 mg/kg/d), methotrexate (0.5 mg/kg s.c. once weekly) and etanercept (25 mg s.c. once weekly) was initiated. After one month, the prednisone was reduced to 30 mg/day. Due to the inability to taper the corticosteroids below 15 mg/day, etanercept was switched to anakinra (100 mg s.c. daily) and intravenous immunoglobulins (2 g/kg) after 3 months. Because of persistent clinical activity and pain at the injection sites, cytokine blockade was switched from anakinra to infliximab one month later, and methylprednisolone infusions were resumed. At the end of this month, the patient developed swelling and increasing pain of the lower back. She had gained 11.5 kg, and her body mass index (BMI) had increased from 20.7 (79^th ^percentile) to 23.6 (95^th ^percentile), respectively, by this time. In addition to fluid along the myofascial border and persisting bilateral sacroiliitis, an MRI of the spine and back demonstrated marked epidural lipomatosis involving spinal segments T1-S5 (Figure 1). Assuming that the lipomatosis was the cause of the worsening back pain, methylprednisolone infusions were discontinued and prednisone was tapered from 30 mg daily to 10 mg daily over a 4-week period. Indeed, these changes gradually lead to a complete remission of the back pain without worsening of the polychondritis. A follow up MRI was not performed because of this dramatic improvement. The patient remains free of back pain at 15 months of follow up.

### Analysis of the literature

PubMed, Web of Science, Google Scholar and the Chinese National Knowledge Infrastructure [CNKI (available at http://www.cnki.net)] were searched for the terms "lipomatosis AND [spine OR spinal OR epidural]". The identified reports were screened manually for patients fulfilling inclusion criteria (corticosteroid treatment, age of onset of lipomatosis before the 18^th ^birthday) and for additional reports. Sixteen cases were identified and pooled with the 3 cases published by us recently [17]. Together with the presented new case, 20 cases were thus available for analysis.

## Results

**Figure 2:**
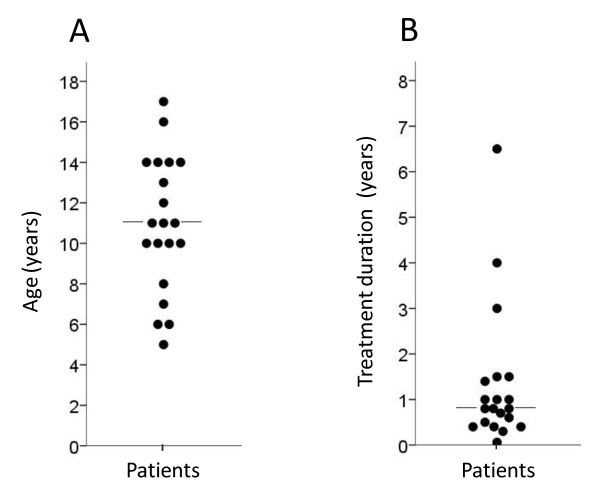
**Dot plots illustrating the spread of age (A) and duration of corticosteroid treatment (B) at the time of diagnosis of epidural lipomatosis**. Values were obtained from the review of the 19 published cases and the presented case (Table 1). Each dot represents the value from one patient. Horizontal lines, median.

Results of the literature analysis and the presented case are summarized in Tables 1 and 2 and Figure 2. The average age at diagnosis of epidural lipomatosis was 11 (+/-3.4) years (SD) (median, 11; range, 5-17) (Figure 2a). Eleven patients (55%) were boys. Corticosteroids were given for nephrotic syndrome in 6 cases (33%), after organ transplantation in 4 cases (20%), for juvenile idiopathic arthritis in 3 cases (15%), for systemic lupus erythematosus in 2 cases (10%), and for one case each of Crohn disease, Sjögren syndrome, Henoch-Schönlein purpura, pineoblastoma, and relapsing polychondritis (presented case). Epidural lipomatosis was diagnosed after a mean 1.3 (+/-1.5) years (SD) (median, 0.8 years; range, 3 weeks - 6.5 years) of corticosteroid treatment. Most cases (17/20) presented within the first 18 months of treatment, and one case (patient 19) after only 3 weeks (Figure 2b). Back pain was among the presenting symptoms in nearly all patients (16 patients, 80%) and was the only documented symptom in 8 patients (40%). A neurological deficit was documented in 10 patients (50%) at the time of diagnosis, with lower extremity weakness being the most common one. The diagnosis was made by MRI in all patients published since 1996. Imaging revealed that the lipomatosis almost always involved the thoracic spine. Lumbosacral and/or cervical spine involvement usually resulted from extension of lipomatosis that also involved the thoracic spine. Preferential involvement of the lumbosacral spine occurred in only two cases (9 and 17), and isolated cervical involvement was not reported. Surgical decompression was performed in only 5 cases (25%), all of which were published before 1997. In the other cases, reduction of corticosteroids (combined with a low-carbohydrate and/or low-fat diet and caloric restriction in 4 cases) sufficed to reduce lipomatosis-associated symptoms. In several patients, corticosteroids had to be tapered despite persisting activity of the underlying medical condition. In patient 14 this was facilitated by increasing the cyclosporine A dose [13], in patients 16 and 17 by stepping up disease-modifying therapy by introducing B cell depletion with rituximab [17], in patient 19 by adding the immunosuppressant everolimus [14], and in the presented case by switching to a different tumor necrosis factor-α blocker. The outcome was good in all cases, except for pt. 4 and 12 who died from progression of a malignancy and from sepsis, respectively, shortly after the lipomatosis was diagnosed. Patient 18 experienced remission of the lipomatosis but died from macrophage activation syndrome 1 year later. Importantly, permanent neurological lesions were not reported in any of the cases and did definitively not develop in patients 16-18 and 20, all of whom are known to the authors of this report.

**Table 1:** Demographic and clinical characteristics of the patients.

**Pt**.	Age (years)	Sex	Diagnosis	Duration of steroid treat- ment (years)	Steroid dose (mg/d)	Therapy	Clinical outcome^a^	Reference
1	17 (?)^b^	m	Kidney transplant	1	40	Surgery SR	Improvement	Lee 1975 [1]
2	13	m	Kidney transplant	1.5	45	SR, diet	Resolution	George 1983 [5]
3	6	m	JIA	4	40	Surgery SR	Improvement	Perling 1988 [6]
4	11	m	Pineoblastoma	1	20	Surgery	Progression of symptoms due to spread of tumor	Quint 1988 [7]
5	6	m	sJIA	1	10-40	Surgery	Resolution	Arroyo 1988 [8]
6	16	m	Kidney transplant	3	0.4/kg	SR, diet	Resolution	Vazquez 1988 [9]
7	10	m	NS	0.7	60	SR, diet	Improvement	Shirai 1990 [10]
8	11	m	NS	0.8	12-60	Surgery	Improvement	Kano 1996 [15]^c^
9	14	f	NS	0.25	24-80	SR	Resolution	-„-
10	14	m	NS	0.4	48-80	SR	Resolution	-„-
11	10	m	HSP	0.8	36-72	SR	Resolution	-„-
12	8	f	Crohn disease	6.5	10-60?	SR	Died from sepsis	Muňoz 2002 [11]
13	14	m	SLE	0.8	<60	SR	Improvement	Miller 2002 [12]
14	5	f	NS	1.4	5-60	SR	Resolution	Kano 2004, 2005 [13,16]
15	10	f	NS	0.4	20-60	SR	Resolution	Kano 2005 [16]
16	14	f	SLE	0.5	0.2/kg	SR	Resolution	Möller 2010 [17]
17	11	f	Sjögren syndrome	0.6	0.5/kg	SR	Resolution	Möller 2010 [17]
18	7	f	sJIA	1.5	40	SR	Resolution^d^	Möller 2010 [17]
19	12	f	Lung transplant	0.06	25	SR, diet	Resolution	Caruba 2010 [14]
20	10	f	Relapsing polychondritis	0.4	30	SR	Resolution	This report

^a^Improvement or resolution as interpreted from the authors' reports of symptoms, findings or imaging.

^b^Age could not be determined accurately, but was more likely 17 than 18 years.

^c^Patient diagnosed in 1979.

^d^Died from chronic macrophage activation syndrome 1 year later.

Abbreviations: f, female; HSP, Henoch Schönlein purpura; LE, lower extremity; m, male; n/d, not done or not documented; NS, nephrotic syndrome; sJIA, systemic juvenile idiopathic arthritis; SLE, systemic lupus erythematosus; SR, steroid reduction; UE, upper extremity.

**Table 2:** Symptoms and neurological findings.

**Pt**.	Symptoms	Documented neurological findings	Approximate extent of lipomatosis	Imaging modality	**Ref**.
1	Weakness/numbness of LE	Motor deficit LE > UE, dysesthesia LE & front of trunk	T1-T11	Myelogram	[1]
2	Hip & low back pain, LE weakness	LE motor deficit; ankle clonus, Babinski sign	T1-L5	Myelogram, CT	[5]
3	LE & thorax pain; loss of function of LE, bowel, and bladder	Paraplegia; sensory deficit below T6, poor anal sphincter tone	T2-T6	Metrimazide CT myelogram	[6]
4	LE weakness, paresthesia, bowel/bladder dysfunction	Sensory deficit below T2-T3	T3-T9	MRI	[7]
5	Back pain, paraplegia	Weak tendon reflexes, sensory deficit to T6	T6-T7*	CT	[8]
6	Upper back and chest pain, paraplegia	Flaccid paresis, Babinski sign, absent abdominal reflexes, decreased LE sense of vibration	T1-T12	CT	[9]
7	Back pain, incontinence to urine, paraplegia	LEs flaccid paresis, Babinski sign	T11-L2/3*	Myelogram, MRI	[10]
8	Back pain, leg weakness	Paraplegia	T1-12*	CT myelogram	[15]
9	Lumbago		L3-S1*	MRI	[15]
10	Lumbago, mid-thoracic back pain, pain with walking		T4-8*, L4-S1*	MRI	[15]
11	Numbness	Cutaneous sensory deficit	T2-6*	MRI	[15]
12	Back pain, gait disturbance	Sensory deficit to T6-T7, UE strength 4/5, LE strength 1/5, absent knee & ankle DTRs, Babinski sign, absent abdominal reflexes	Entire spine	MRI	[11]
13	Back pain	Neurological exam normal	T1-L3+**	MRI	[12]
14	Back pain		T4-S1	MRI	[13,16]
15	Back pain		T7-T9	MRI	[16]
16	Back pain, incontinence to stool and urine, paresthesias	Increased DTRs LE, loss of anal sphincter tone	T2- L5+**	MRI	[17]
17	Back pain, radicular pain		L4-5*	MRI	[17]
18	Low back pain		T2-S4/5	MRI	[17]
19	Right LE weakness, left LE paresthesia, dysuria		T2-T11	MRI	[14]
20	Low back pain	Normal neurologic exam	T1-S5	MRI	This report

*Extent of cord compression

**Lipomatosis extended caudal to L5; exact extent not documented.

Abbreviations: CT, computerized tomography; DTR; deep tendon reflex; LE, lower extremity; MRI, magnetic resonance imaging; n/d, not documented; UE, upper extremity.

## Discussion

The presented case illustrates the general features of epidural lipomatosis that were identified with the literature analysis: onset of symptoms in the context of a pronounced iatrogenic Cushing syndrome --even after a relatively short duration of corticosteroid treatment--, back pain as the most common presenting symptom, and a good neurological outcome without surgical intervention. Therefore, new onset or worsening back pain in a patient on chronic corticosteroid treatment should be warning signs of epidural lipomatosis. Considering the documented value of MRI in the early detection of epidural lipomatosis [15], a spinal MRI should be performed when these warning signs are present. A marked increase in BMI, due to a corticosteroid-induced Cushing syndrome, had taken place in our patient by the time the lipomatosis manifested. In the literature analysis it was not possible to calculate BMIs before initiation of corticosteroid treatment and at the time of diagnosis in a sufficient number of cases. However, features of a Cushing syndrome were documented in most of the cases, thus supporting the notion that corticosteroid-associated epidural lipomatosis represents a variant outcome of iatrogenic Cushing syndrome. As substantiated by the clinical improvement in our patient and by the results of the literature analysis, reducing corticosteroid dosing should be the first-line intervention when epidural lipomatosis is detected. However, it may be difficult to do this without compromising treatment of the underlying chronic illness. As documented in patients 12, 16, 17, 19 and 20, intensifying the disease-modifying treatment regimen with a potentially steroid-sparing agent should be considered strongly. In addition, in patients 16 and 17 prednisone was discontinued in favor of deflazacort, a corticosteroid that may have similar immunosuppressive effectiveness as prednisone but lower glucocorticoid side effects [18]. The role of adjunct dietary interventions geared toward mobilizing fat deposits remains to be defined better. In summary, epidural lipomatosis represents a rare but potentially serious complication of chronic corticosteroid treatment, which, if diagnosed in a timely manner, can be treated effectively with tapering corticosteroids.

It should be pointed out that this analysis of published cases is subject to the well known pitfalls of publication bias. Thus, relatively straight forward cases with a good outcome are likely under reported and may be more common than can be gleaned from our analysis. The authors of this report would therefore welcome input from colleagues around the world who are interested in formulating larger and prospective studies relating to corticosteroid-induced epidural lipomatosis in the pediatric age group.

## Competing interests

The authors declare that they have no competing interests.

## Authors' contributions

JM participated in data collection and writing the manuscript. RQC contributed the case report and edited the manuscript. DWY contributed the MR image and its radiographic interpretation. HJG, DL, DDS, AK, and KS participated in data collection and edited the manuscript. FP initiated and oversaw the study, participated in writing the manuscript and is responsible for its final editing. He had access to all data and takes responsibility for the integrity of the data. All authors read and approved the final manuscript.

## Consent

Consent for publication of this report was obtained from the patient and her parents and is available for review by the editors of *Pediatric Rheumatology*.
